# Gastric epithelial neoplasm of fundic-gland mucosa lineage: proposal for a new classification in association with gastric adenocarcinoma of fundic-gland type

**DOI:** 10.1007/s00535-021-01813-z

**Published:** 2021-07-15

**Authors:** Hiroya Ueyama, Takashi Yao, Yoichi Akazawa, Takuo Hayashi, Koichi Kurahara, Yumi Oshiro, Masayoshi Yamada, Ichiro Oda, Shin Fujioka, Chiaki Kusumoto, Masayoshi Fukuda, Kunihisa Uchita, Tomohiro Kadota, Yasuhiro Oono, Kazuhisa Okamoto, Kazunari Murakami, Yasumasa Matsuo, Motohiko Kato, Tadateru Maehata, Naohisa Yahagi, Yumiko Yasuhara, Tomoyuki Yada, Koji Uraushihara, Tetsumi Yamane, Taiji Matsuo, Masanori Ito, Yasuhiko Maruyama, Ayumi Osako, Shoko Ono, Mototsugu Kato, Kazuyoshi Yagi, Takashi Hashimoto, Natsumi Tomita, Sho Tsuyama, Tsuyoshi Saito, Kohei Matsumoto, Kenshi Matsumoto, Sumio Watanabe, Naomi Uemura, Tsutomu Chiba, Akihito Nagahara

**Affiliations:** 1 Department of Gastroenterology, Juntendo University School of Medicine, 2-1-1 Hongo, Bunkyo-Ku, Tokyo, 113-8421 Japan; 2 Department of Human Pathology, Juntendo University Graduate School of Medicine, Tokyo, Japan; 3 Department of Human Pathology, Juntendo University School of Medicine, Tokyo, Japan; 4 Department of Gastroenterology, Matsuyama Red Cross Hospital, Ehime, Japan; 5 Department of Pathology, Matsuyama Red Cross Hospital, Ehime, Japan; 6 Endoscopy Division, National Cancer Center Hospital, Tokyo, Japan; 7 Division of Gastroenterology, Fukuoka Red Cross Hospital, Fukuoka, Japan; 8 Department of Gastroenterology, Nippon Kokan Fukuyama Hospital, Hiroshima, Japan; 9 Department of Gastroenterology and Hepatology, Tokyo Medical and Dental University, Tokyo, Japan; 10 Department of Gastroenterology, Kochi Red Cross Hospital, Kochi, Japan; 11 Department of Gastroenterology and Endoscopy, National Cancer Center Hospital East, Chiba, Japan; 12 Department of Gastroenterology, Faculty of Medicine, Oita University, Oita, Japan; 13 Division of Gastroenterology and Hepatology, Department of Internal Medicine, St. Marianna University School of Medicine, Kanagawa, Japan; 14 Division of Gastroenterology and Hepatology, Department of Internal Medicine, Keio University School of Medicine, Tokyo, Japan; 15 Division of Research and Development for Minimally Invasive Treatment, Cancer Center, Keio University School of Medicine, Tokyo, Japan; 16 Department of Diagnostic Pathology, Kyoto-Katsura Hospital, Kyoto, Japan; 17 Department of Gastroenterology & Hepatology, National Center for Global Health and Medicine, Kohnodai Hospital, Chiba, Japan; 18 Department of Gastroenterology and Hepatology, Showa General Hospital, Tokyo, Japan; 19 Department of Diagnostic Pathology, Tottori Red Cross Hospital, Tottori, Japan; 20 Department of Endoscopy, Hiroshima University Hospital, Hiroshima, Japan; 21 Department of General Internal Medicine, Hiroshima University Hospital, Hiroshima, Japan; 22 Department of Gastroenterology, Fujieda Municipal General Hospital, Shizuoka, Japan; 23 Department of Gastroenterology, Tottori Seikyo Hospital, Tottori, Japan; 24 Department of Gastroenterology, Hokkaido University Hospital, Hokkaido, Japan; 25 Department of Gastroenterology, National Hospital Organization Hakodate National Hospital, Hokkaido, Japan; 26 Department of Gastroenterology and Hepatology, Uonuma Institute of Community Medicine, Niigata University Medical and Dental Hospital, Niigata, Japan; 27 Department of Esophageal and Gastroenterological Surgery, Juntendo University School of Medicine, Tokyo, Japan; 28 Department of Gastroenterology and Hepatology, Kyoto University Graduate School of Medicine, Kyoto, Japan; 29 Kansai Electric Power Hospital, Osaka, Japan

**Keywords:** Gastric adenocarcinoma of fundic-gland type, Gastric adenocarcinoma of fundic-gland mucosa type, Gastric epithelial neoplasm of fundic-gland mucosa lineage, Oxyntic gland adenoma

## Abstract

**Background:**

Gastric adenocarcinoma of fundic-gland type (GA-FG) is a rare variant of gastric neoplasia. However, the etiology, classification, and clinicopathological features of gastric epithelial neoplasm of fundic-gland mucosa lineage (GEN-FGML; generic term of GA-FG related neoplasm) are not fully elucidated. We performed a large, multicenter, retrospective study to establish a new classification and clarify the clinicopathological features of GEN-FGML.

**Methods:**

One hundred GEN-FGML lesions in 94 patients were collected from 35 institutions between 2008 and 2019. We designed a new histopathological classification of GEN-FGML using immunohistochemical analysis and analyzed via clinicopathological, immunohistochemical, and genetic evaluation.

**Results:**

GEN-FGML was classified into 3 major types; oxyntic gland adenoma (OGA), GA-FG, and gastric adenocarcinoma of fundic-gland mucosa type (GA-FGM). In addition, GA-FGM was classified into 3 subtypes; Type 1 (organized with exposure type), Type 2 (disorganized with exposure type), and Type 3 (disorganized with non-exposure type). OGA and GA-FG demonstrated low-grade epithelial neoplasm, and GA-FGM should be categorized as an aggressive variant of GEN-FGML that demonstrated high-grade epithelial neoplasm (Type 2 > 1, 3). The frequent presence of *GNAS* mutation was a characteristic genetic feature of GEN-FGML (7/34, 20.6%; OGA 1/3, 33.3%; GA-FG 3/24, 12.5%; GA-FGM 3/7, 42.9%) in mutation analysis using next-generation sequencing.

**Conclusions:**

We have established a new histopathological classification of GEN-FGML and propose a new lineage of gastric epithelial neoplasm that harbors recurrent *GNAS* mutation. This classification will be useful to estimate the malignant potential of GEN-FGML and establish an appropriate standard therapeutic approach.

**Supplementary Information:**

The online version contains supplementary material available at 10.1007/s00535-021-01813-z.

## Introduction

We previously proposed gastric adenocarcinoma of fundic-gland type (GA-FG) as a new form of gastric adenocarcinoma with distinct clinicopathological and endoscopic features [1–5]. GA-FG is defined by a well-differentiated adenocarcinoma composed of pale gray-blue, basophilic columnar cells with mild nuclear atypia, resembling chief cells that are positive for immunohistochemical staining of pepsinogen-I (a marker of chief cells) and/or H + K + -ATPase (a marker of parietal cells). This subtype represents a novel type of gastric cancer that is not associated with *H. pylori* infection [1, 2]. In 2017, GA-FG was added as a special cancer type to the Japanese classification of gastric carcinoma [6]. In addition, GA-FG was also listed as a new and rare gastric neoplasia in the WHO Classification of Tumors, 2019 [7]. GA-FG is expected to account for an increasing proportion of all gastric cancers, since *H. pylori* infection have been significantly reduced due to eradication therapy and improved public health.

With regard to biological behavior, GA-FG is considered less aggressive because it exhibits low cellular atypia, no vascular invasion, low proliferative activity, a lack of p53 protein overexpression, and good prognosis [1]. Therefore, some previous reports on GA-FG have preferred the terms “oxyntic gland polyp/adenoma”, “chief cell-predominant gastric polyps” or “oxyntic gland adenoma (OGA)”, to describe lesions which were generally viewed as benign [8–10]. OGA was also listed as a new rare and benign gastric epithelial neoplasm composed of columnar cells with differentiation to chief cells, parietal cells, or both, with a high rate of progression to adenocarcinoma (submucosal invasion) in the WHO Classification of Tumors, 2019 [7]. When the tumor shows invasion to the submucosa, it should be classified as GA-FG. However, there is ongoing discussion as to whether OGA should be regarded as an intramucosal phase of GA-FG.

Recently, some cases of an aggressive variant of GA-FG with high cellular atypia have been discovered [11–17]. This variant exhibited differentiation toward gastric foveolar epithelium in addition to fundic-gland differentiation, and it was designated as ‘gastric adenocarcinoma of fundic-gland mucosa type’ (GA-FGM) [13, 18]. Based on the components of cell differentiation, those of GA-FG that were the same as normal fundic-gland mucosa have been named GA-FGM. However, the definition, classification, and clinicopathological features of GA-FGM are poorly described. Most Japanese pathologists who are specialized in gastroenterological pathology believe that gastric epithelial neoplasm of fundic-gland mucosa lineage (GEN-FGML; generic term of GA-FG related neoplasm) may be classified histopathologically into GA-FG and GA-FGM subtypes, and that GA-FGM has a high malignant potential. However, previous reports have relied on a small number of GEN-FGML cases to define the clinicopathological and genetic features of this cancer [11–18]. Thus, the definition, classification, and clinicopathological features of GEN-FGML remain vague and fragmentary. To address this lack of clarity, we designed the current study to establish the definition and classification of GEN-FGML and elucidate the clinicopathological features of GEN-FGML. This was achieved by analyzing a large number of GEN-FGML cases, which has allowed us to estimate the malignant potential of this disease and establish a standard therapeutic approach for its treatment.

## Materials and methods

### Samples and tissue collection

A total of 149 GEN-FGMLs obtained consecutively between January 2008 and December 2019 were collected from 35 institutions and analyzed retrospectively. Of 149 lesions, 100 lesions in 94 patients that could be evaluated in detail clinically, histopathologically, and immunohistochemically were included in this study. The 20 cases analyzed in our previous reports were also included in this study [1, 2]. GEN-FGML was defined as a gastric epithelial neoplasm with a differentiated adenocarcinoma resembling fundic-gland cells, which was positively stained for pepsinogen-I and/or H + /K + -ATPase in at least 10% of the tumor.

### Histopathological classification of GA-FGML

**Fig. 1:**
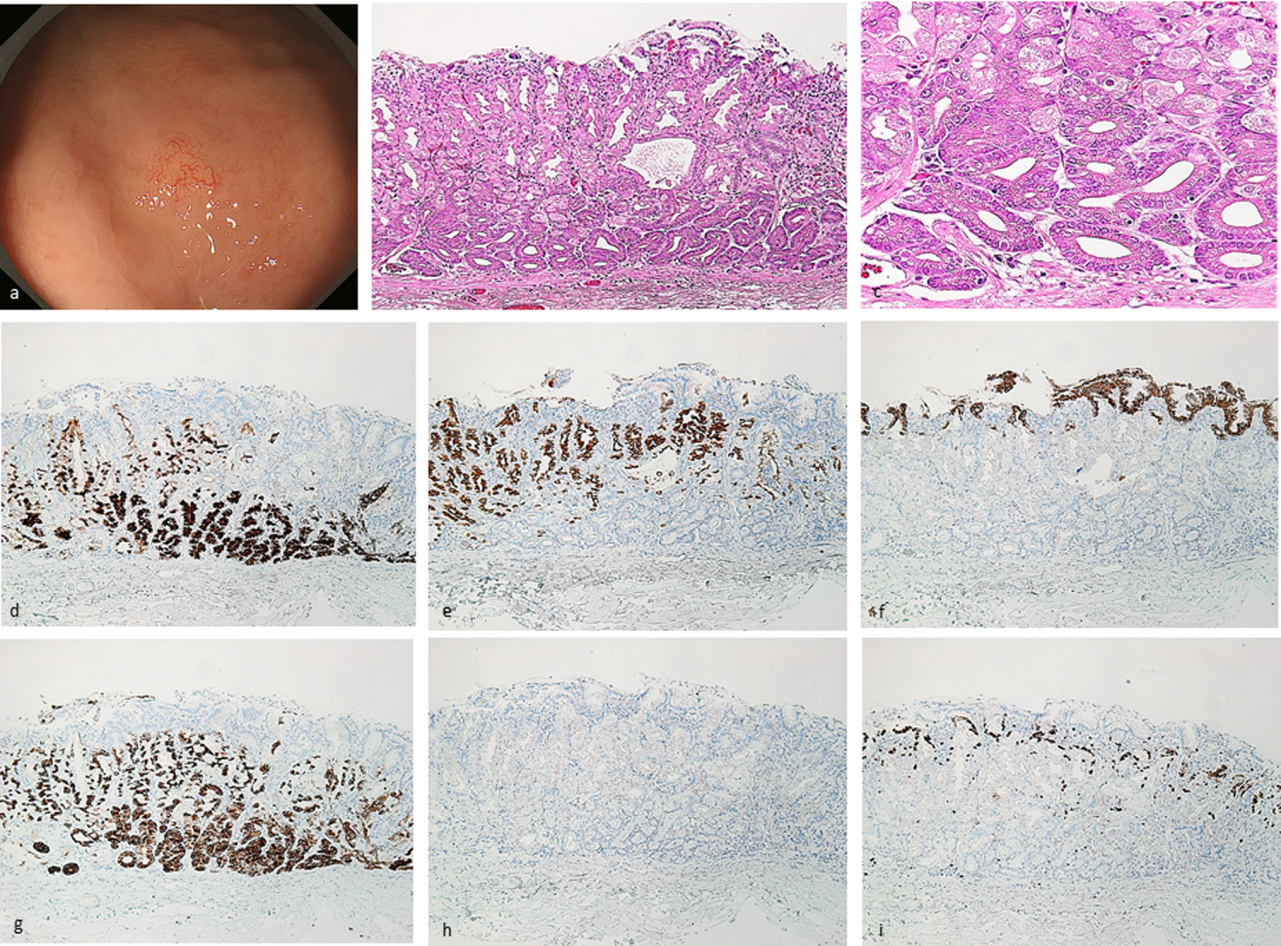
Oxyntic gland adenoma. Endoscopic image by white light endoscopy; **a** The flatly elevated lesion with submucosal tumor shape was located at the greater curvature of the fornix and had a whitish color and dilated vessels with branch architecture. The background mucosa had no atrophic change. Histological features (**b**, **c**); **b** Oxyntic gland adenoma was located at only deep area. The surface of the lesion was covered with normal foveolar epithelium. **c** The tumor cells were mainly composed of highly differentiated columnar cells mimicking fundic gland cells, predominantly chief cells, with pale gray-blue, basophilic cytoplasms, and mildly enlarged nuclei. Immunohistochemical results (**d**–**i**); **d** pepsinogen-I ( +), **e** H + /K + -ATPase (focally +), **f** MUC5AC (−, MUC5AC is only positive for superficial non-neoplastic foveolar cells), **g** MUC6 ( +), **h** p53 (−), **i** Ki-67 Labeling Index = 1%. Pathological diagnosis; U, 0-IIb, 5 × 4 mm, Oxyntic gland adenoma, UL0, Ly0, V0, HM0, VM0

**Fig. 2:**
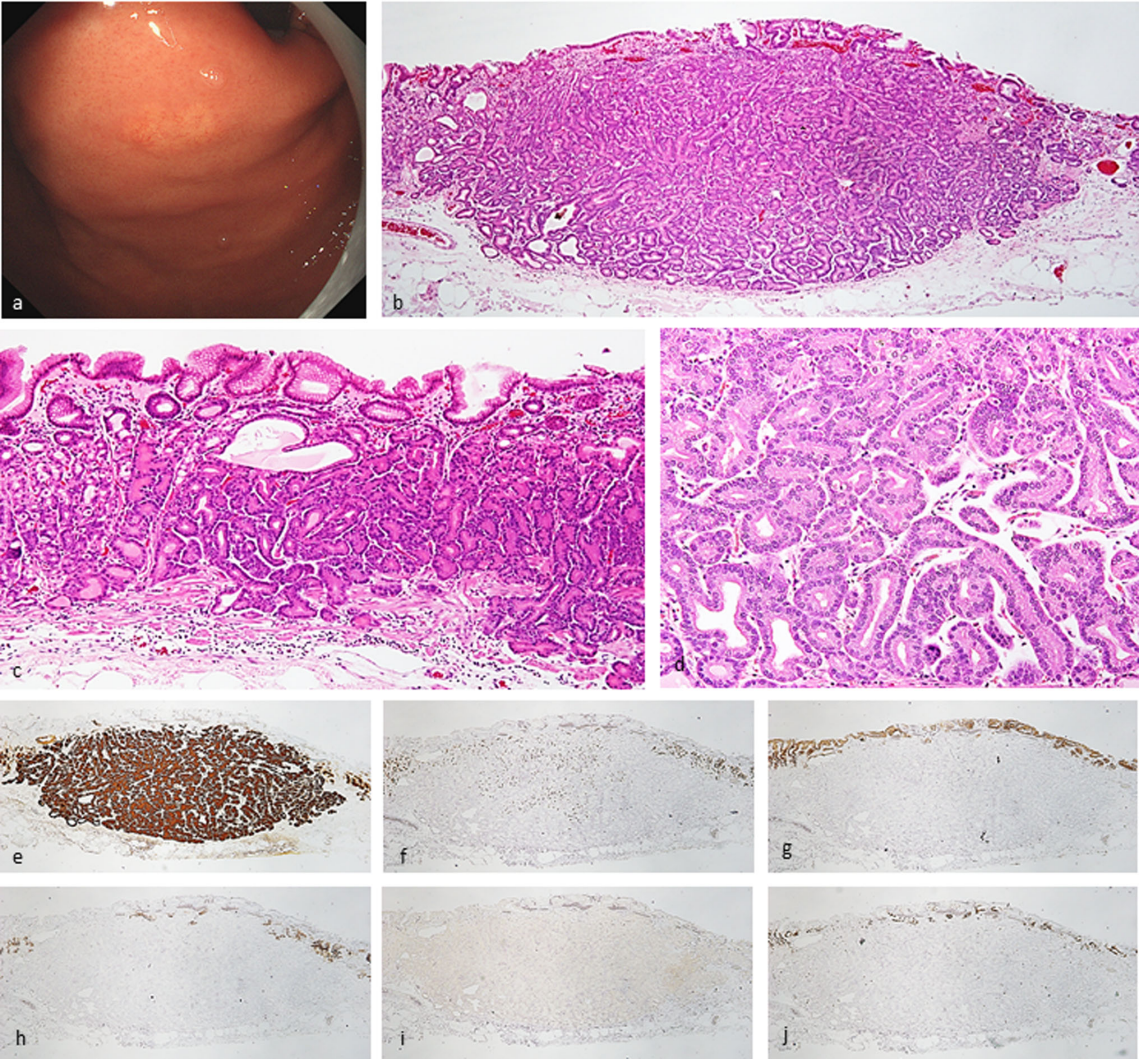
Gastric adenocarcinoma of fundic-gland type. Endoscopic image by white light endoscopy; **a** The flatly elevated lesion with submucosal tumor shape was located at the greater curvature of the cardia and had a whitish color and dilated vessels with branch architecture. The background mucosa had no atrophic change. Histological features (**b**–**d**); **b**, **c** Gastric adenocarcinoma resembling fundic-gland cells was located from beneath surface to deep area (SM600μm). The surface of the lesion was covered with normal foveolar epithelium, whereas the deep area of the tumor showed irregular branching and dilatation. **d** The tumor cells were mainly composed of highly differentiated columnar cells mimicking fundic-gland cells, predominantly chief cells, with pale gray-blue, basophilic cytoplasms, and mildly enlarged nuclei. Immunohistochemical results (**e**–**j**); **e** pepsinogen-I ( +), **f** H + /K + -ATPase (focally +), **g** MUC5AC (−), **h** MUC6 (−), **i** p53 (−), **j** Ki-67 Labeling Index = 1%. Pathological diagnosis; U, 0-IIa, 9 × 6 mm, gastric adenocarcinoma of fundic-gland type, pT1b/SM2(600 µm), UL0, Ly0, V0, HM0, VM0

**Fig. 3:**
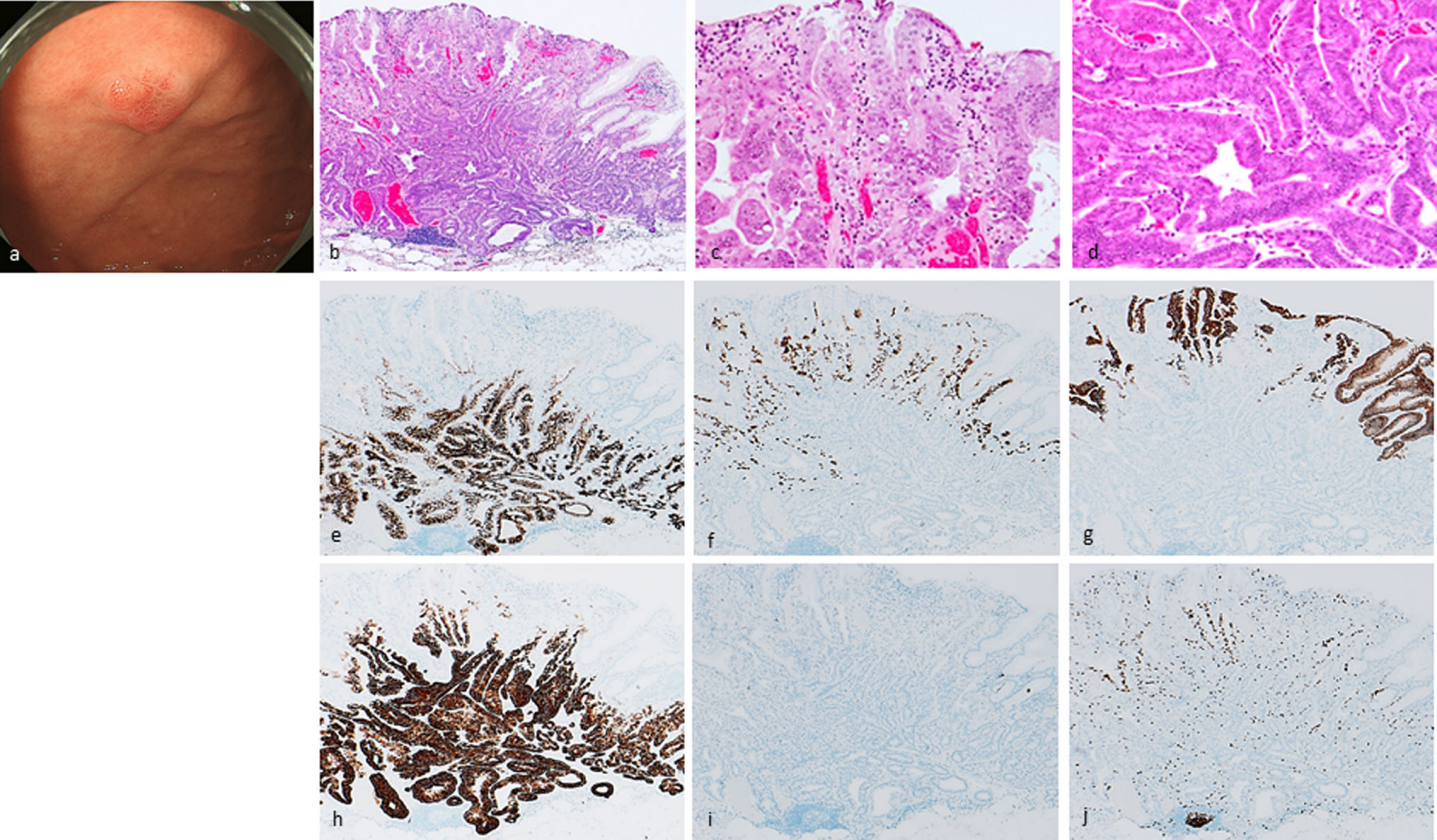
Gastric adenocarcinoma of fundic-gland mucosa type: type 1 (organized with exposure type). Endoscopic image by white light endoscopy; **a** The flatly elevated lesion with submucosal tumor shape was located at the posterior wall side of the fornix and had a reddish color. The background mucosa had no atrophic change. Histological features (**b**−**d**); **b** Gastric adenocarcinoma resembling fundic-gland cells was located at deep area (SM700μm) and showed irregular branching and dilatation. The surface area of the lesion was composed of foveolar type well-differentiated adenocarcinoma followed by the component of GA-FG. Tissue construct of foveolar epithelium and fundic gland is maintained, and tumor is exposed on the surface. **c** (surface area) The tumor cells were composed of foveolar type well-differentiated adenocarcinoma with low-grade atypia. **d** (deep area) The tumor cells were mainly composed of highly differentiated columnar cells mimicking fundic-gland cells, predominantly chief cells, with pale gray-blue, basophilic cytoplasms, and mildly enlarged nuclei. Immunohistochemical results (**e**−**j**); **e** pepsinogen-I ( +), **f** H + /K + -ATPase (focally +), **g** MUC5AC (surface, +), **h** MUC6 ( +), **i** p53 (−), **j** Ki-67 Labeling Index = 10%. Pathological diagnosis; U, 0-IIa, 15 × 13 mm, gastric adenocarcinoma of fundic-gland mucosa type, pT1b/SM2(700 µm), UL0, Ly0, V0, HM0, VM0

**Fig. 4:**
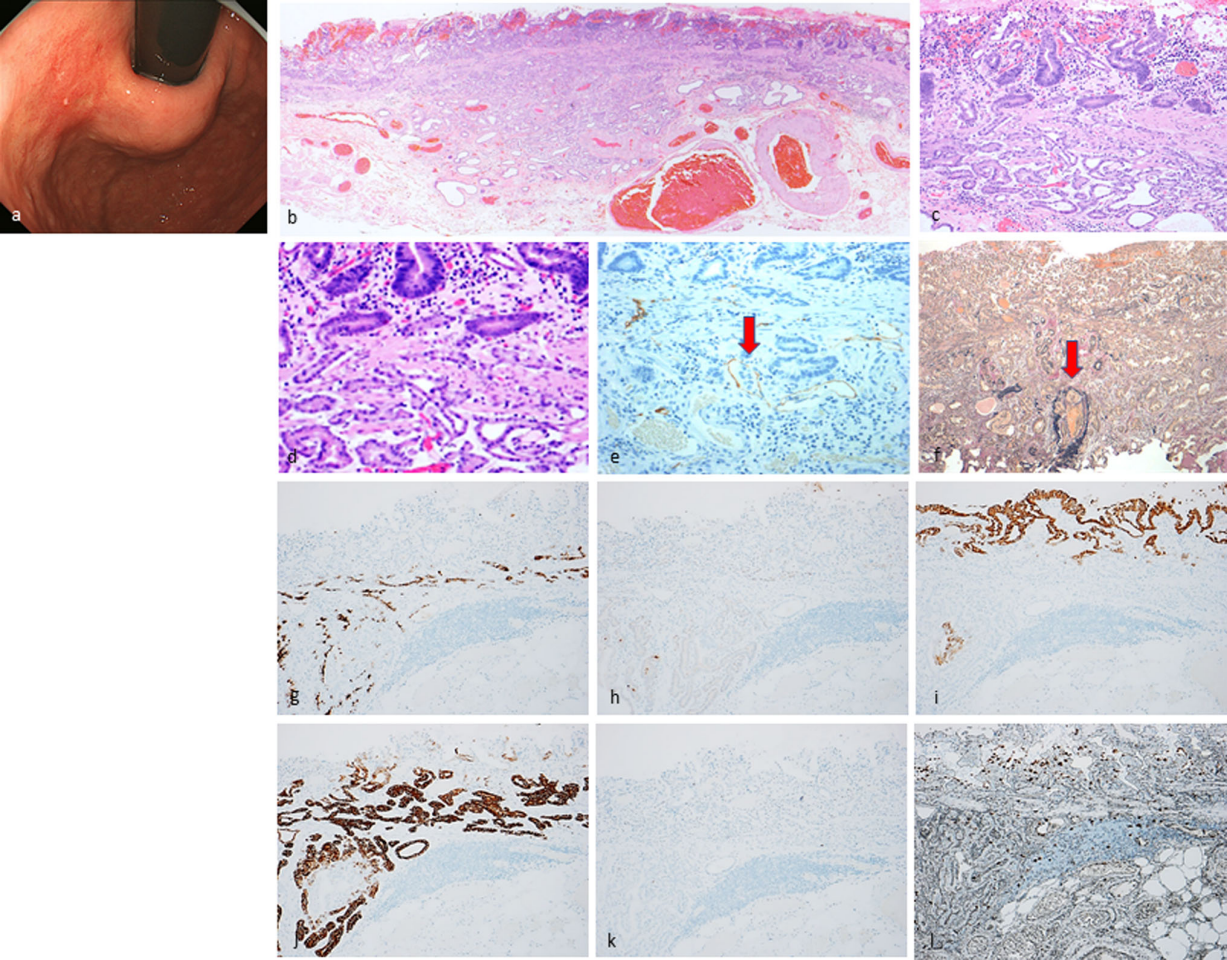
Gastric adenocarcinoma of fundic-gland mucosa type: type 2 (disorganized with exposure type). Endoscopic image by white light endoscopy; **a** The reddish depressed lesion was located at the posterior wall side of the cardia. The background mucosa had no atrophic change. Histological features (**b**–**d**); **b**, **c**, **d** Gastric adenocarcinoma resembling fundic-gland cells was located at deep area (SM1000μm) and showed irregular branching and dilatation. The surface area of the lesion was composed of foveolar type well-differentiated adenocarcinoma with low-grade atypia. However, the layered architecture was destroyed, and tissue construct of foveolar epithelium and fundic gland is collapsed, and tumor is exposed on the surface. Immunohistochemical/histochemical results (**e**–**l**); **e** Lymphatic invasion (red arrow) demonstrated by D2-40, **f** Venous invasion (red arrow) demonstrated by EVG, **g** pepsinogen-I ( +), **h** H + /K + -ATPase (focally +), **i** MUC5AC (surface and deep area +), **j** MUC6 ( +), **k** p53 (−), **l** Ki-67 Labeling Index = 10%). Pathological diagnosis; U, 0-IIc, 25 × 20 mm, Adenocarcinoma of fundic-gland mucosa type, T1b/SM2(1000 µm), UL1, Ly1, V1, pHM0, pVM0

**Fig. 5:**
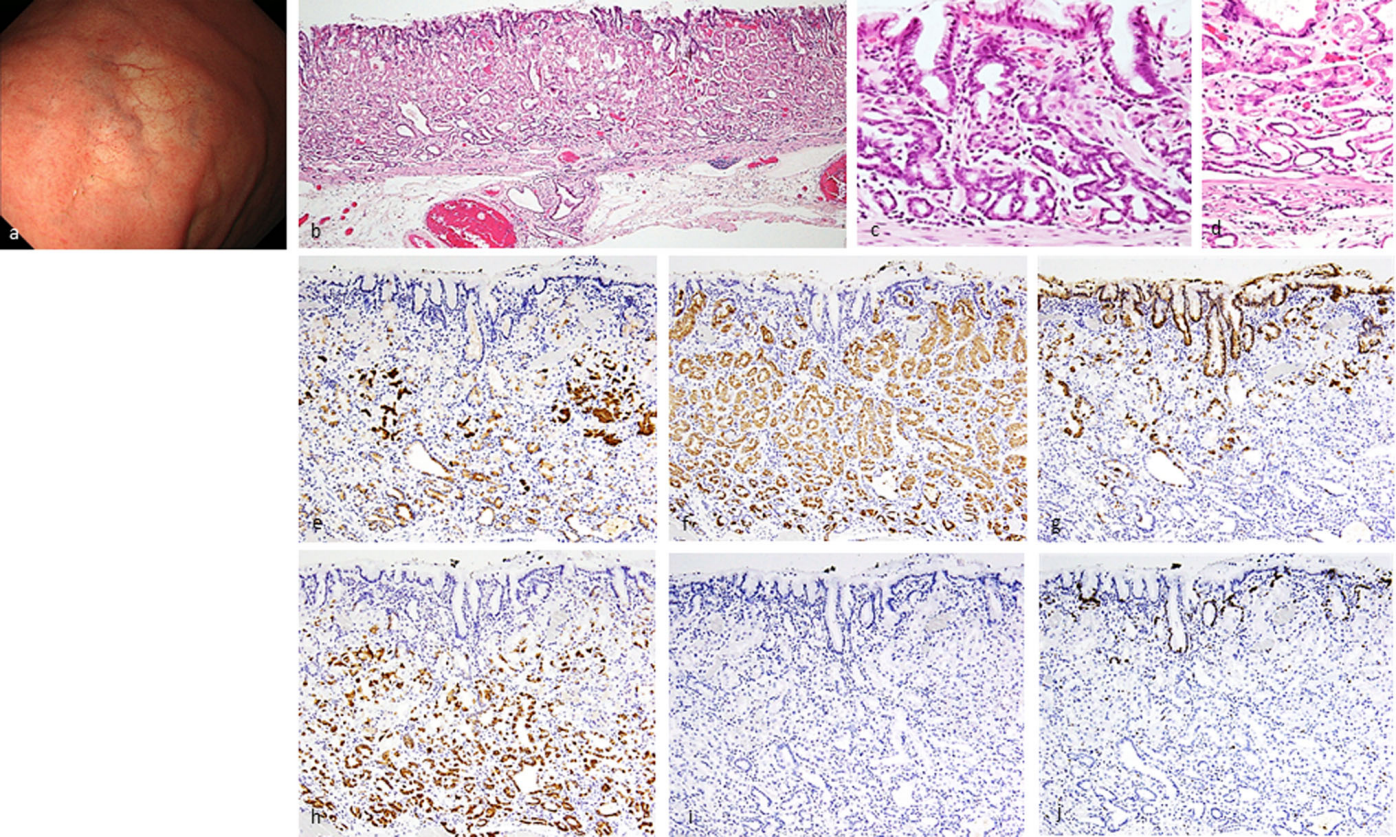
Gastric adenocarcinoma of fundic-gland mucosa type: Type 3 (disorganized with non-exposure type). Endoscopic image by white light endoscopy; **a** The flatly elevated lesion with submucosal tumor shape was located at the greater curvature of the cardia and had a whitish color and dilated vessels with branch architecture. The background mucosa had no atrophic change. Histological features (**b**–**d**); **b**, **c** Gastric adenocarcinoma resembling fundic-gland cells was located at deep area and showed irregular branching and dilatation. The surface of the lesion was covered with normal foveolar epithelium. **d** In the middle of the mucosal layer, the tumor cells were composed of highly differentiated columnar cells mimicking fundic-gland cells, predominantly parietal cells. Immunohistochemical results (**e**–**j**); **e** pepsinogen-I ( +), **f** H + /K + -ATPase (diffuse +), **g** MUC5AC ( +), **h** MUC6 ( +), **i** p53 (−), **j** Ki-67 Labeling Index = 3%). The tumor cells exhibited differentiation both gastric foveolar epithelium and fundic-gland differentiation in the deep area. Tissue construct of foveolar epithelium and fundic gland is collapsed, and tumor is not exposed on the surface. Pathological diagnosis; U, 0-IIc, 27 × 23 mm, Adenocarcinoma of fundic-gland mucosa type, T1b/SM1(100 µm), UL0, Ly0, V0, pHM0, pVM0

All lesions were classified immunohistochemically by the combination of MUC5AC (a marker for gastric foveolar epithelial cells), MUC6 (a marker for gastric mucous neck cells), pepsinogen-I, and H + /K + -ATPase. The immunohistochemical classification of GEN-FGML is shown in Supplementary Table 1. According to this classification and some previous reports [1, 7, 13, 18], GEN-FGML can be classified into 3 major types as follows; OGA, GA-FG and GA-FGM. Both OGA and GA-FG are composed of proliferation of fundic gland-like cells in irregular glands [MUC5AC-, MUC6 + or -, pepsinogen-I and/or H + K + -ATPase +]. OGA is defined by mucosal neoplasia (Fig. 1), whereas GA-FG is defined by submucosal invasive neoplasia (Fig. 2). GA-FGM is defined by neoplasia composed of a mixture of fundic gland-like and foveolar-like cells [MUC5AC + , MUC6 + , pepsinogen-I and/or H + K + -ATPase +] (Figs. 3, 4, 5). OGA and GA-FG are usually covered by non-neoplastic mucosa. Although OGA and GA-FG are rarely accompanied by an exposed tumor component, the exposed tumor component is less than 10% of the tumor. In addition, according to the mucosal architecture of foveolar epithelium and the fundic-gland, GA-FGM can be further classified into 3 subtypes as follows. Type 1 (organized with exposure type): the normal histological architecture of fundic-gland mucosa (superficial foveolar differentiation (MUC5AC is positive at superficial area) and deeper fundic-gland differentiation) is preserved; Type 2 (disorganized with exposure type): the abnormal histological architecture is demonstrated by aberrant expression of MUC5AC (MUC5AC is positive not only at superficial area but also at deeper area) and the tumor is exposed on the surface, and Type 3 (disorganized with non-exposure type): the abnormal histological architecture is demonstrated by aberrant expression of MUC5AC (MUC5AC is positive at deeper area), and the tumor is covered by non-neoplastic mucosa (Supplementary Fig. 3). “Exposure”, and not “erosion”, means that the tumor is exposed on the surface. The presence or absence of tumor exposure is a very important finding when considering the histological architecture of the tumor. All histology and immunohistochemical staining results were evaluated by one pathologist (Yao T) who is specialized in the pathophysiology of the gastrointestinal tract and is one of the authors of the OGA section in WHO Classification of Tumors, 2019. Immunohistochemical examination and evaluation, and phenotypic classification were described in the Supplementary Materials.

**Table 1:** Clinicopathological characteristics, immunohistochemical analysis, and *GNAS* mutation of OGA, GA-FG, and GA-FGM

	OGA (*n* = 20)	GA-FG (*n* = 55)	GA-FGM (*n* = 25)	*p* value
Clinicopathological characteristics				
Sex (male: female)	12: 8	33: 22	22: 3	0.07
Age (average: years)	66.4 (range: 47–80)	66.9 (range: 38–87)	66.1(range: 40–85)	0.93
Therapy	ESD: EMR: OPE = 17: 3: 0	ESD: EMR: OPE = 45: 6: 4	ESD: EMR: OPE = 15: 2: 8	< 0.05
Location	U: M: L = 15: 5: 0	U: M: L = 46: 9: 0	U: M: L = 18: 5: 2	0.60
Morphological classification	Protruded: flat/depressed = 13: 7	Protruded: flat/depressed = 37: 18	Protruded: flat/depressed = 12: 13	0.37
Size of tumor (average: mm)	7.1 (range: 2–18)	8.4 (range: 1.5–43)	20.8 (range: 4–85)	< 0.01
Depth of invasion (μm)	NA	251.5 (50–1400) µm	M: SM = 4: 21 1212.5 (100–4750) µm	< 0.01
Lymphatic invasion	0%, 0/20	0%, 0/55	24%, 6/25	< 0.01
Venous invasion	0%, 0/20	1.8%, 1/55	20%, 5/25	< 0.05
Horizontal margin	5%, 1/20	1.8%, 1/55	16%, 4/25	0.14
Vertical margin	0%, 0/20	1.8%, 1/55	8%, 2/25	0.67
Lymph node metastasis	NA	0%, 0/2	7.7%, 1/13	0.26
*H.pylori* infection	( +): 2, (−): 13, (Eradication): 3	( +): 5, (−): 28, (Eradication): 9	( +): 2, (−): 11, (Eradication): 1	0.94
Survival time (average: months)	20.9 (range: 1–105) (20 cases)	28.6 (range: 1–113) (44 cases)	16.1 (range: 1–30) (18 cases)	0.87
Outcome	20 cases: alive NED	43 cases: alive NED, one case died of CVD	18 cases: alive NED	NA
Immunohistochemical analysis				
pepsinogen-I	100%, 20/20	100%, 55/55	100%, 25/25	0.99
H^+^/K^+^-ATPase (> focally +)	82.4%, 14/17	83.7%, 41/49	73.9%, 17/23	0.79
MUC2	0%, 0/20	0%, 0/51	4.3%, 1/23	0.72
MUC5AC	0%, 0/20	0%, 0/55	100%, 25/25	< 0.01
MUC6	100%, 20/20	98.2%, 54/55	100%, 25/25	0.70
CD10	0%, 0/20	0%, 0/51	4.3%, 1/23	0.72
Phenotypic classification	G: GI = 20: 0	G: GI = 51: 0	G: GI = 21: 2	0.29
Chromogranin-A (focally +)	0%, 0/13	0%, 0/29	7.1%, 1/14	0.75
p53 overexpression (focally +)	0%, 0/20	2.0%, 1/51	21.1%, 4/19	< 0.05
Ki-67 MIB1 LI (%)	6.8% (18 cases)	4.7% (47 cases)	9.9% (21 cases)	0.06
*GNAS* mutation	33.3%, 1/3	12.5%, 3/24	42.9%, 3/7	0.46

*OGA* oxyntic gland adenoma, *GA-FG* gastric adenocarcinoma of fundic-gland type, *GA-FGM* gastric adenocarcinoma of fundic-gland mucosa type, *ESD* endoscopic submucosal dissection, *EMR* endoscopic mucosal resection, *OPE* operation, *U* upper third of the stomach, *M* middle third of the stomach, *L* lower third of the stomach, *NA*, not assessed, *M* intramucosal cancer, *SM* submucosal cancer, *NED* no evidence of disease, *CVD* cardiovascular disease, *G* gastric phenotype, *GI* gastrointestinal phenotype

### Mutation analysis and next-generation sequencing

All resected specimens underwent a uniform preparation protocol for formalin-fixed paraffin-embedded (FFPE) specimens. Tumoral and corresponding non-tumoral FFPE samples were collected from each patient, and genomic DNA was extracted using the QIAamp FFPE tissue kit (Qiagen, Antwerp, Belgium). We performed next-generation sequencing (NGS) using the Ion Ampliseq Cancer Hotspot Panel v2 (Thermo Fisher Scientific, Waltham, MA, USA, Supplementary Fig. 2) for 34 lesions in Juntendo University School of Medicine; samples were selected based on the assessment of DNA integrity. Detailed procedures for molecular analyses are described in the Supplementary Materials. We evaluated the molecular characteristics of GEN-FGML in comparison with conventional gastric adenocarcinoma. The genetic alterations identified by NGS were compared with the database of The Cancer Genome Atlas (TCGA, Firehose Legacy; www.cbioportal.org).

### Evaluation method

We established a new classification of GEN-FGML [OGA, GA-FG, and GA-FGM (Type 1, Type 2, and Type3)] and compared OGA, GA-FG, and GA-FGM cases, and 3 subtypes of GA-FGM via clinicopathological, immunohistochemical, and genetic evaluation (OGA vs. GA-FG vs. GA-FGM and Type 1 vs. 2 vs. 3). In addition, we evaluated whether OGA should be regarded as an intramucosal phase of GA-FG.

### Statistical analysis

All statistical analyses were performed with EZR (Easy R; Saitama Medical Center, Jichi Medical University, Saitama, Japan) [19], which is a graphical user interface for R (The R Foundation for Statistical Computing, Vienna, Austria). Continuous data were compared with the Mann–Whitney *U* test. Categorical analysis of variables was performed using the Fisher exact test. A *p* value < 0.05 was considered to indicate a statistically significant difference.

### Ethics

This study was conducted in accordance with the Declaration of Helsinki and approved by the Institutional Review Board of Juntendo University School of Medicine (approval number: #14–166, #16–104) and participating institutions which have provided the clinicopathological and immunohistochemical data for this research. Patients were not required to give consent for the study because the analysis used anonymous clinical data that were obtained after each patient agreed to treatment by verbal and documental consent. Individuals cannot be identified from the data presented.

## Results

Based on the new classification of GEN-FGML, 100 GEN-FGML lesions were subclassified into 20 OGAs, 55 GA-FGs, and 25 GA-FGMs [Type 1 (*n* = 11): organized with exposure type; Type 2 (*n* = 10): disorganized with exposure type; Type 3 (*n* = 4): disorganized with non-exposure type] (Supplementary Fig. 3).

### Oxyntic gland adenoma and gastric adenocarcinoma of fundic-gland type demonstrated low-grade epithelial neoplasms

Twenty lesions were categorized as OGA (Table 1, Fig. 1) and fifty-five lesions were categorized as GA-FG (Table 1, Fig. 2). The majority of OGA and GA-FG presented as flat elevated lesions with a submucosal tumor shape, located in the upper-to-middle third of the stomach and had a whitish color and dilated vessels with a branching architecture. The background mucosa exhibited no atrophic changes. However, reddish or flat/depressed lesions were also observed. Regarding color and morphology, OGA and GA-FG were divided into four groups in the descending order of frequency as follows: whitish and protruded type > whitish and flat or depressed type > reddish and protruded type > reddish and flat or depressed type. Although GA-FG tumors invaded into the SM layer, they had small diameters (average: 8.4 mm) in the absence of lymphatic invasion (0/55, 0%), vascular invasion (1/55, 1.8%), and lymph node metastasis (0/2, 0%). The rate of *H. pylori* infection was low, however, some cases were positive or post eradication (positive: negative (uninfected): post eradication = 7: 41: 12). Histopathologically, the tumor cells were mainly composed of highly differentiated columnar cells mimicking fundic-gland cells (predominantly chief cells) with pale gray-blue, basophilic cytoplasms, and mildly enlarged nuclei. In some lesions, the tumor cells with coarse granular eosinophilic cytoplasms were admixed and were similar to parietal cells. The surface of the lesion was covered with normal foveolar epithelium, whereas the deeper regions of the tumor showed irregular branching and dilatation. Marked desmoplastic reaction was absent from lesions in the invasive area. Immunohistological staining analysis showed that all lesions were positive for pepsinogen-I (75/75, 100%), and most lesions were focally positive for H + /K + -ATPase (55/66, 83.3%). As for the mucin phenotype, all lesions presented with a gastric phenotype. Only one lesion exhibited focal p53 overexpression. The mean Ki-67/MIB1 labeling index (LI) was 5.3%. We considered this rate to be low, and the positive cells were irregularly distributed without formation of a proliferative zone. In surrounding non-neoplastic mucosa, the positive cells were regularly distributed with formation of a proliferative zone (mucous neck cell). There was no significant difference in histopathological features between OGA and GA-FG except for the depth of invasion. The frequency of *GNAS* mutation in OGA and GA-FG was 33.3% (1/3) and 14.8% (4/27), respectively.

### Gastric adenocarcinoma of fundic-gland mucosa type demonstrated high-grade epithelial neoplasms

Twenty-five lesions were categorized as GA-FGM (Table 1, Figs. 3, 4, 5). Most of these lesions were located in the upper-to-middle third of the stomach, and only two were detected in the lower third. Macroscopically, there were slightly more flat/depressed lesions than there were protruded lesions. Regarding color and morphology, GA-FGM was divided into four groups in the descending order of frequency as follows: reddish and protruded type > whitish and flat/depressed type > reddish and flat/depressed type > whitish and protruded type. The mean tumor diameter of GA-FGM lesions was 20.8 mm. GA-FGM was highly invasive into the SM layer (21/25, 84%) and was accompanied by vascular invasion (8/25, 32%); only one case showed lymph node metastasis (1/13, 7.7%) (Supplementary Fig. 4). The rate of *H. pylori* infection was low, however, some cases were positive or post eradication (positive: negative (uninfected): post eradication = 2: 11: 1). Histopathologically, GA-FGM exhibited differentiation toward gastric foveolar epithelium (MUC5AC-positive) and mucous neck cells or pyloric gland-like (MUC6-positive) mucous cells; fundic-gland differentiation was also present. As for the ratio of tumor components, differentiation toward gastric foveolar epithelium was superior to fundic-gland differentiation. Compared to the GA-FG lesions, GA-FGM lesions had a higher degree of histological atypia; furthermore, some cases were accompanied with the common type of differentiated adenocarcinoma. Immunohistological staining analysis showed that all lesions were positive for pepsinogen-I, MUC5AC and MUC6 (25/25, 100%), and most lesions were focally positive for H + /K + -ATPase (17/23, 73.9%). However, some cases revealed highly differentiated columnar cells mimicking fundic-gland cells; these were predominantly parietal cells rather than chief cells (Fig. 3). As for the mucin phenotype, most GA-FGM were gastric in nature; however, only 2 cases showed a gastrointestinal phenotype. Four cases exhibited focal p53 overexpression (4/19, 21.1%), and the mean Ki-67 MIB1 LI was 9.9%. Twenty-one cases of GA-FGM (21/25, 84.0%) had foveolar type tumor (MUC5AC-positive) on the surface (and were therefore ‘exposure’ type lesions). Four cases were covered with non-neoplastic foveolar epithelium on the tumor and the deeper area of the lesion was composed of various tumor cells (MUC5AC-positive) (the ‘non-exposure type’, Type 3, Fig. 5). Foveolar type tumors exposed on the surface tended to exhibit low-grade histological atypia (Figs. 3, 4). However, the neoplastic epithelium can be histologically identified by the careful observation of cytological (slightly enlarged nuclei, hyperchromasia, and prominent nucleoli) and architectural features (tortuous glands, branching glands, and anastomosing glands) with the presence of an abrupt transition between the atypical and the non-neoplastic epithelium. The frequency of *GNAS* mutation in GA-FG was 42.9% (3/7).

### Clinicopathological characteristics of GEN-FGML

Supplementary Table 2 shows the clinicopathological characteristics of all the 100 cases of GEN-FGML. Of 16 patients who underwent surgery including additional surgical resection, lymph node metastasis was found only in one case of Type 2 GA-FGM (6.3%) (Supplementary Fig. 4). Among 77 patients treated using the endoscopic procedure, 15 were regarded as non-curative resection cases according to the Japanese Gastric Cancer Treatment Guideline 2018 (5th edition) [20]. Half of the total number of non-curative resection cases were Type 2 GA-FGM (Supplementary Table 3). Eleven of 15 cases were available for the follow-up survey; 3 cases underwent additional surgical resection. During the follow-up period, there was no recurrence, metastasis, or gastric cancer-specific death in all the 11 cases, including the 8 cases in which additional surgical resection was not performed.

**Table 2:** Clinicopathological characteristics, immunohistochemical analysis, and *GNAS* mutation of GA-FGM (Type 1, Type 2, and Type 3)

	Type 1 (*n* = 11)	Type 2 (*n* = 10)	Type 3 (*n* = 4)	*p* value
Clinicopathological characteristics				
Sex (male: female)	10: 1	8: 2	4: 0	0.94
Age (average: years)	64.4 (range: 40–85)	67.3 (range: 41–83)	68 (range: 43–77)	0.85
Therapy	ESD: EMR: OPE = 6: 2: 3	ESD: EMR: OPE = 7: 0: 3	ESD: EMR: OPE = 2: 0: 2	0.94
Location	U: M: L = 8: 2: 1	U: M: L = 7: 2: 1	U: M: L = 3: 1: 0	0.95
Morphological classification	Protruded: flat/depressed = 7: 4	Protruded: flat/depressed = 4: 6	Protruded: flat/depressed = 1: 3	0.94
Size of tumor (average: mm)	11.6 (4–30)	31.8 (14–85)	18.5 (5–31)	< 0.05
Depth of invasion (μm)	M: SM = 3: 8 657.1 (200–1200) µm	M: SM = 0: 10 1460 (400–4000) µm	M: SM = 1: 3 1683.3 (100–4750) µm	0.52 0.42
Lymphatic invasion	0%, 0/11	60%, 6/10	0%, 0/4	< 0.05
Venous invasion	9.1%, 1/11	30%, 3/10	25%, 1/4	0.75
Horizontal margin	9.1%, 1/11	20%, 2/10	0%, 0/4	0.94
Vertical margin	0%, 0/11	20%, 2/10	0%, 0/4	0.62
Lymph node metastasis	0%, 0/3	14.3%, 1/7	0%, 0/3	0.71
*H.pylori* infection	( +): 0, (-): 8, (Eradication): 1	( +): 0, (-): 2, (Eradication): 1	( +): 1, (-): 0, (Eradication): 0	0.46
Survival time (average: months)	11.9 (range: 1–47) (10 cases)	21.8 (range: 1–47) (6 cases)	19.5 (range: 15–24) (2 cases)	0.48
Outcome	10 cases: Alive NED	6 cases: Alive NED	2 cases: Alive NED	
Immunohistochemical analysis				
pepsinogen-I	100%, 11/11	100%, 10/10	100%, 4/4	0.95
H^+^/K^+^-ATPase (> focally +)	60%, 6/10	77.8%, 7/9	100%, 4/4	0.67
MUC2	9.1%, 1/11	0%, 0/9	0%, 0/3	0.57
MUC5AC	100%, 11/11	100%, 10/10	100%, 4/4	0.95
MUC6	100%, 11/11	100%, 10/10	100%, 4/4	0.95
CD10	0%, 0/11	11.1%, 1/9	0%, 0/3	0.57
Phenotypic classification	G: GI = 10: 1	G: GI = 8: 1	G: GI = 3: 0	0.74
Chromogranin-A (focally +)	0%, 0/5	0%, 0/6	33.3%, 1/3	0.78
p53 overexpression (focally +)	14.3%, 1/9	42.9%, 3/7	0%, 0/3	0.59
Ki-67 MIB1 LI (%)	12.1% (9 cases)	10% (7 cases)	4.8% (4 cases)	0.36
*GNAS* mutation	50%, 3/6	0%, 0/1	NA	0.88

*GA-FGM* gastric adenocarcinoma of fundic-gland mucosa type, *ESD* gastric adenocarcinoma of fundic-gland mucosal type, *ESD* endoscopic submucosal dissection, *EMR* endoscopic mucosal resection, *OPE* operation, *U* upper third of the stomach, *M* middle third of the stomach, *L* lower third of the stomach, *NA* not assessed, *M* intramucosal cancer, *SM* submucosal cancer, *NED* no evidence of disease, *CVD* cardiovascular disease, *G* gastric phenotype, *GI* gastrointestinal phenotype

The clinicopathological characteristics of OGA, GA-FG, and GA-FGM lesions are shown in Table 1. There were more males (*p* = 0.07) and more cases in which surgical resection was performed as the first treatment method (*p* < 0.05) in GA-FGM than in OGA and GA-FG. The average tumor size (OGA 7.1 mm vs. GA-FG 8.4 mm vs. GA-FGM 20.8 mm, p < 0.01) and depth of submucosal invasion (GA-FG 251.5 μm vs. GA-FGM 1212.5 μm, *p* < 0.01) were significantly greater in GA-FGM than in OGA and GA-FG. Furthermore, the rates of lymphatic and venous invasion were significantly higher in GA-FGM than in OGA and GA-FG (Ly: 0 vs. 0 vs. 24%, *p* < 0.01, V: 0 vs. 1.8 vs. 20%, *p* < 0.05). There was no significant difference in *H. pylori* infection status among OGA, GA-FG, and GA-FGM. Immunohistochemically, GA-FGM included two cases with a gastrointestinal phenotype; this subtype also had a higher frequency of p53 overexpression (*p* < 0.05) and higher Ki-67/MIB1 LI (*p* = 0.06) than OGA and GA-FG.

The clinicopathological characteristics of Type 1, 2, and 3 are shown in Table 2. The average tumor size (Type 1: 11.6 mm vs. Type 2: 31.8 mm vs. Type C-3: 18.5 mm, *p *< 0.05) was significantly greater and the rates of lymphatic invasion (0 vs. 60 vs. 0%, *p* < 0.05) were significantly higher in Type 2 than in Types 1 and 3. There was no significant difference in *H. pylori* infection status among Type 1, 2, and 3.

NGS analysis revealed that 19 of 34 GEN-FGML patients (55.9%) had oncogenic mutations including *GNAS* (7/34, 20.6%), *KRAS* (2/34, 5.9%), *PIK3CA* (2/34, 5.9%), and *CDKN2A* (1/34, 2.9%) (Supplementary Table 4). No tumors harbored *TP53* mutations. The frequency of *GNAS* mutation in the three major subtypes was as follows: OGA (1/3, 33.3%), GA-FG (3/24, 12.5%), and GA-FGM (3/7, 42.9%). There were no other nonsynonymous and type-specific mutations identified in these tumors. Among 34 cases, 2 tumors harbored multiple oncogenic alterations: *GNAS* co-occurred with *CDKN2A* mutations in GA-FG, and *GNAS* co-occurred with *PIK3CA* mutations in GA-FGM, respectively; both were classified as non-curative resection following an endoscopic procedure. Compared with public data sets of conventional gastric adenocarcinoma (TCGA, Firehose Legacy), the frequency of *GNAS* mutation was significantly higher in GEN-FGML (7/34, 20.6% vs. 21/395, 5.3%, *p* < 0.01) whereas the frequency of *TP53* mutation was significantly lower in GEN-FGML (0/34, 0% vs. 190/395, 48.1%, p < 0.01). However, there were no significant differences in the frequencies of *KRAS* mutation (2/34, 5.9% vs. 37/395, 9.4%, *p* = 0.71), *PIK3CA* mutation (2/34, 5.9% vs. 65/395, 16.5%, *p* = 0.17) or *CDKN2A* mutation (1/34, 2.9% vs. 17/395, 4.3%, *p* = 0.95).

Overall comparison between OGA and GA-FG revealed that (with the exception of tumor invasion depth) there were no significant differences in clinicopathological, endoscopic, and molecular-biological findings (Table 1, Supplementary Table 4).

## Discussion

Our study is the first comprehensive analysis of GEN-FGML and has the largest number of GEN-FGML cases when compare to previous reports. We established a new histopathological classification of GEN-FGML as follows: OGA, GA-FG, and GA-FGM (Type 1, 2, and 3) (Supplementary Fig. 3). We classified OGA and GA-FG clinicopathologically as low-grade gastric epithelial neoplasms, which is consistent with previous reports. However, we classified GA-FGM (and especially Type 2) as a high-grade gastric epithelial neoplasm. In regard to the molecular pathological concept of GEN-FGML, *GNAS* mutation may be a characteristic genetic feature of GEN-FGML, and OGA, GA-FG, and GA-FGM may prove to be distinctive gastric neoplasia of the same lineage related to *GNAS* mutation.

Although many GEN-FGML cases have since been reported in both Japan and other countries, they were mainly presented in case reports or series and reviews; to date, a comprehensive analysis has not been reported [1–5, 8–18, 21–33]. Moreover, there have only been tentative classifications of GEN-FGML proposed so far, which were devised by Japanese pathologists specialized in gastroenterological pathology [5, 13, 18]. In the present study, we placed a high priority on the classification of GEN-FGML according to cell differentiation rather than histological atypia to develop a standard classification for facilitating its widespread use. Relying on classification based on histological atypia can make analysis of tumorigenesis difficult due to the mixture differentiated cells; this can also lead to subjective evaluations by individual pathologists. Therefore, we established the new classification of GEN-FGML based on cell differentiation and histological architecture. Although hematoxylin and eosin (H&E) staining provides some information on cell differentiation, we chose a more accurate immunohistochemical method that will allow standardized tumor analysis.

GA-FGM is occasionally accompanied by tumor components consisting of mucous neck cells or pyloric gland-like (MUC6-positive mucous cell) differentiation. Therefore, GA-FGM presumably possess multilineage potential. GA-FGM should be categorized as a distinct type of complex gastric adenocarcinoma accompanied by components exhibiting fundic-gland differentiation. Meanwhile, when a GEN-FGML lesion is continuously accompanied with the common type of gastric adenocarcinoma consisting of differentiation toward gastric foveolar epithelium (MUC5AC-positive), it is also diagnosed as GA-FGM. Such lesions should be considered as variants of Type 2 GA-FGM, since the etiology and carcinogenesis of such a lesion may differ from common-type gastric adenocarcinoma due to mixtures of existing components exhibiting fundic-gland differentiation. These cases are high-grade malignancies, [16, 17] as also shown in this study. Type 2 GA-FGM had highest malignant potential among all GEN-FGML subtypes due to the high associated vascular invasion rate and non-curative resection rate, and the possibility of lymph node metastasis (Supplementary Fig. 4). Gastroenterologists and pathologists should pay great attention to the high malignant potential of GA-FGM based on this histopathological classification, and the possibility that GA-FGM cases could be missed if they are simply labeled as common-type gastric adenocarcinomas. When the cytological features of the mucosal component of adenocarcinomas are similar to those of fundic-gland cells, and fundic-gland differentiation is confirmed by immunohistochemical staining (pepsinogen-I and H + K + -ATPase), the lesion can be classified as GA-FGM. Furthermore, the detection of the GNAS mutation might support the diagnosis of GA-FGM.

According to the WHO classification, intramucosal lesions are listed as benign OGA. However, OGAs and GA-FG lesions could be considered the same adenocarcinoma, since they share features in terms of endoscopic, histological, immunohistochemical, and molecular-biological aspects, and only differ with regard to the depth of invasion. Reports from countries other than Japan argued that oxyntic gland neoplasms might show prolapse-type misplacement rather than true submucosal invasion [8]. In some cases, the lack of desmoplastic reaction may suggest prolapse-type misplacement. However, differentiated adenocarcinoma with low-grade atypia can invade into the submucosal layer without evoking a desmoplastic reaction, and thus such lesions should be considered as true invasion rather than prolapse-type misplacement, even if the histological atypia is low-grade [1, 34]. This confusion is derived from the fact that Japan has used different histopathological criteria from the United States and European nations for determining whether intramucosal lesions of gastric adenocarcinoma with low-grade atypia are intramucosal carcinomas or benign tumors. Intramucosal lesions of gastric adenocarcinoma with low-grade atypia should be diagnosed as carcinoma in Japan, based on the fact that they can invade into the submucosal layer and are accompanied with vascular invasion while maintaining the grade of histological atypia [34]. Therefore, so-called OGA, at least for a lesion presenting with histological features similar to GA-FG, should be regarded as an intramucosal phase of GA-FG.

The endoscopic features of OGA and GA-FG revealed a flat elevated lesion with a submucosal tumor shape, located in the upper-to-middle third of the stomach, without atrophy, and with a whitish color and dilated vessels with a branching architecture, as in our previous reports [2, 3]. Conversely, the endoscopic features of GA-FGM revealed a reddish lesion and there were slightly more flat/depressed lesions than there were protruded lesions. Therefore, it may be possible to endoscopically differentiate OGA and GA-FG from type 1 and 2 GA-FGM according to color and morphology, due to the difference in components in the superficial area. However, the endoscopic differentiation of OGA and GA-FG from Type 3 GA-FGM may be difficult because the surface of the lesion is usually covered by non-neoplastic mucosa. Based on this histopathological classification of GEN-FGML, further investigations, including magnifying endoscopy analysis, are required to elucidate the endoscopic features of GEN-FGML.

This histopathological classification based on the result of the present study is useful for malignancy grading of GEN-FGML lesions. This malignancy grading is not only important in carcinogenesis analysis, but it can also be used in the clinic to determine whether additional surgical resection after endoscopic treatment is required. In the curative resection for tumors of expanded indication of the Japanese Gastric Cancer Treatment Guideline 2018 (5th edition) [20], additional surgical resection is indicated for differentiated type gastric cancers when submucosal invasion depth is equal to or greater than 500 μm. Since GA-FG exhibited very few vascular invasions and neither recurrence nor metastasis regardless of SM invasion depth, we suggest that additional surgical resection may not be indicated for such GA-FG cases. Furthermore, there have been some reports of GA-FG cases available for long-term follow-up observation [33]. However, to establish an indication criterion for endoscopic treatment and additional surgical resection of GA-FG, we should provide a 95% confidence interval for the risk of lymph node metastasis in GA-FG and GA-FGM. Therefore, we should compare the risk of surgical resection and discuss the possible measures for avoiding additional surgical resection in GA-FG cases in the future. On the other hand, since GA-FGM is more malignant than GA-FG, endoscopic treatment guidelines for common-type gastric adenocarcinoma should be applied to GA-FGM cases. At this moment, however, treatment of any of GA-FGML cases should fully comply with the guidelines established for common-type gastric adenocarcinoma. In the future, long-term follow-up analysis with a large number of GEN-FGML cases will be needed to establish a standard therapeutic approach for each type of GEN-FGML. This will help to define the criteria for endoscopic curative resection specific to GEN-FGML, as well as help to elucidate the natural history of GEN-FGML.

GEN-FGML involves alterations in genes associated with *Wnt/β-catenin* signaling pathways, *GNAS* mutation, *KRAS* mutation, and the *sonic hedgehog* signaling pathway [18, 35–41]. We have detected mutations in *CTNNB1*, *AXINs*, *APC*, or *GNAS* in approximately half of GEN-FGML cases, and suggest that activation of the *Wnt/β-catenin* signaling pathway might have a key role in carcinogenesis in these malignancies [35–38]. In the present study, GEN-FGML cases were classified into OGA, GA-FG, and GA-FGM subtypes, and gene mutations were comprehensively analyzed using NGS technology. Compared with public data sets of conventional gastric adenocarcinoma (TCGA, Firehose Legacy), the frequency of *GNAS* mutation was significantly higher in GEN-FGML (7/34, 20.6% vs. 21/395, 5.3%, *p* < 0.01). Since the frequency of *GNAS* mutation in common-type gastric adenocarcinoma is very low, we suggest that *GNAS* mutation was a characteristic genetic feature of GEN-FGML. Furthermore, we could demonstrate that all three types of GEN-FGML belong to the same genetic lineage, since cases of all three types displayed *GNAS* mutation. The frequency of *KRAS* mutation was 8.6% (3/35), which is not significantly different from public data sets (37/395, 9.4%) (*p* = 0.88). However, *KRAS* mutation may be relevant to GEN-FGML etiology with the same frequency as common-type gastric adenocarcinoma. No *TP53* mutation have been detected so far in our analyses, and there was only localized immunohistochemical staining in a few cells, suggesting that GA-FGM is a lower-grade malignancy than common-type gastric adenocarcinoma. In future, evaluation of genomic, transcriptomic, and epigenetic changes will be needed to fully elucidate the malignant alterations of GEN-FGML.

We suggest that GA-FG may progress to GA-FGM eventually by malignant alteration of the histological atypia, switching differentiation status, and gaining multilineage potential while maintaining *GNAS* mutation. However, this hypothesis was not fully tested in our current study. We analyzed whether *H. pylori* infection was relevant to malignant alteration of GEN-FGML. There was no significant difference in clinicopathological features (with the exception of age and Ki-67 MIB1 labeling index) and molecular-biological features between *H.pylori* negative group and *H.pylori* positive or eradicated group (Supplementary Tables 4, 5). In any case, further examination will be needed to determine whether *H. pylori* can promote tumor progression in GEN-FGML.

This study has several potential limitations. First, our study was analyzed retrospectively. Second, the evaluation of more cases and longer follow-up would be required to confirm the malignant potential of GEN-FGML and draw accurate conclusions, since there was no difference in the clinical outcomes between OGA, GA-FG, and GA-FGM cases, probably because of the small number of GEN-FGML cases. However, GA-FGM is considered to have a potential for aggressive biological behavior because its rates of lymphatic and venous invasion are significantly higher than those in GA-FG. Third, cases available for NGS analysis were available from only one institution because of the ethical codes surrounding enrollment. Forth, the NGS analysis was performed on genomic DNA extracted by microdissection without lasers, and the cut-off level of allele frequency was set as 3%.

In conclusion, we have developed a novel classification of GEN-FGML into OGA subtypes, GA-FG subtypes, and GA-FGM subtypes (Type 1, 2, and 3). OGA and GA-FG demonstrated low-grade epithelial neoplasm, and OGA should be regarded as an intramucosal phase of GA-FG. GA-FGM should be categorized as an aggressive variant of GEN-FGML that demonstrated high-grade epithelial neoplasm (Type 2 > 1, 3). This classification will be useful to estimate the malignant potential of GEN-FGML and establish an appropriate standard therapeutic approach.

## Supplementary Information

Below is the link to the electronic supplementary material.

Supplementary file1 (DOCX 18 KB)

Supplementary file2 Supplementary Fig. 1 Phenotypic classification (TIF 65 KB)

Supplementary file3 Supplementary Fig. 2 Fifty gene of the Ion Ampliseq Cancer Hotspot Panel v2 (TIF 87 KB)

Supplementary file4 Supplementary Fig. 3 Histopathological classification of GEN-FGML (TIF 107 KB)

Supplementary file5 Supplementary Fig. 4 Gastric adenocarcinoma of fundic-gland mucosa type: Type 2 (disorganized with exposure type) with LN metastasis. Endoscopic image by white light endoscopy; **a** The reddish elevated lesion was located at the greater curvature of the lower third of the stomach. The background mucosa had atrophic change. Histological features (**b**, **e**, **f**, **i**, **j**); **b** The surface area of the lesion was composed of foveolar type well-differentiated adenocarcinoma with low-grade atypia. **b**, **e**. Adenocarcinoma resembling fundic-gland cells was located at deep area and showed irregular branching and dilatation. **f** In the middle of the mucosal layer, the tumor cells were composed of highly differentiated columnar cells mimicking fundic-gland cells. **i**, **j**. LN metastasis was shown at n#4d. Immunohistochemical results (**c** ,**d**, **g**, **h**, **k**, **l**); **c** MUC5AC (surface and deep area +), **d** MUC6 (surface and deep area +), **g** pepsinogen-I (focally +), **h** H+/K+-ATPase (focally +). Lymph node **k** MUC6 (+), **l** pepsinogen-I (focally +).The layered architecture was destroyed, and tissue construct of foveolar epithelium and fundic gland is collapsed, and tumor is exposed on the surface. Pathological diagnosis; L, 0-IIa, 19x15mm, Adenocarcinoma of fundic-gland mucosa type, T1b/SM2, UL0, Ly0, V0, pHM0, pVM0, pN1 (n#4d:1/4) (TIF 1431 KB)

Supplementary file6 (DOCX 13 KB)

Supplementary file7 (DOCX 17 KB)

Supplementary file8 (DOCX 15 KB)

Supplementary file9 (DOCX 20 KB)

Supplementary file10 (DOCX 19 KB)

## Abbreviations

GA-FG: Gastric adenocarcinoma of fundic-gland type
GEN-FGML: Gastric epithelial neoplasm of fundic-gland mucosa lineage
GA-FGM: Gastric adenocarcinoma of fundic-gland mucosa type
OGA: Oxyntic gland adenoma
FFPE: Formalin-fixed paraffin-embedded
NGS: Next generation sequence
