# Supplementary material for: Gastric epithelial neoplasm of fundic-gland mucosa lineage: proposal for a new classification in association with gastric adenocarcinoma of fundic-gland type
Source: J Gastroenterol. 2021 Jul 15;56(9):814–28. doi: 10.1007/s00535-021-01813-z (PMC8370942; doi:10.1007/s00535-021-01813-z)
Supplement: Supplementary file 1 — Supplementary file1 (DOCX 18 KB) [file 535_2021_1813_MOESM1_ESM.docx]

**Supplementary Materials**

**Immunohistochemical Examination and Evaluation**

One representative section from each case was prepared for immunohistochemical staining. We used monoclonal antibodies against MUC5AC (Novocastra, Newcastle-upon-Tyne, UK, diluted 1:100) as a marker for gastric foveolar cells, MUC6 (Novocastra, Newcastleupon- Tyne, UK, diluted 1:100) as a marker for gastric mucous neck cells and pyloric glands, MUC2 (Novocastra, Newcastle-upon-Tyne, UK, diluted 1:100) as a marker for intestinal goblet cells, CD10 (Novocastra, Newcastle-upon-Tyne, UK, diluted 1:100) as a marker for the small intestinal brush border, chromogranin A (DAKO, Glostrup, Denmark, diluted 1:200) as a marker for neuroendocrine cells, pepsinogen-I (AbD Serotec, Oxford, UK, diluted 1:100) as a marker for chief cells, H+/K+-ATPase a subunit (MBL, Nagoya, Japan, diluted 1:500) as a marker for parietal cells, Ki-67 (MIB-1, dilution 1:100; DAKO, Glostrup, Denmark), and p53 (PAb 1801, dilution 1:50; Novocastra, Newcastle- upon-Tyne, UK).

Immunohistochemical staining was carried out using the streptavidin-biotin-peroxidase complex method (Histofine SAB-PO Kit, Nichirei, Tokyo, Japan) after antigen retrieval with microwave heating (citrate buffer, 30 min). Sections were visualized with diaminobenzidine and counterstained with hematoxylin. The negative controls were produced by substituting mouse normal serum for the primary antibodies.

Cell differentiation by MUC2, MUC5AC, MUC6, CD10, pepsinogen-I, H+/K+-ATPase and chromogranin-A, cell proliferation by Ki-67, and overexpression of p53 protein were evaluated immunohistochemically. Tissues were scored positive for MUC2, MUC5AC, MUC6, CD10, pepsinogen-I, H+/K+-ATPase, and chromogranin-A when more than 10% of the area was positively stained. The Ki-67 (MIB-1) labeling index (LI) was defined as the percentage of MIB-1-positive nuclei and was evaluated in the invasive areas. The MIB-1 LI was determined by counting at least 1000 nuclei in the selected fields at x400 magnification. The p53 immunoreactivity was defined as positive when distinct nuclear staining was recognized in at least 10% of the cells, since most of the earlier published studies used this as the cut-off level. Cases with less than 10% positive cells were regarded as negative.

**Phenotypic Classification**

The phenotypes were classified into 4 categories according to the combination of the expression of CD10 (brush border), MUC2 (goblet cells), MUC5AC (gastric foveolar epithelium), and MUC6 (mucous neck cells, pyloric glands). The intestinal (I) phenotype exhibited expression of either CD10 or MUC2 but not of MUC5AC or MUC6. The gastrointestinal (GI) phenotype exhibited expression of either CD10 or MUC2, in addition to expression of either MUC5AC or MUC6. The gastric (G) phenotype exhibited expression of either MUC5AC or MUC6 but not of CD10 or MUC2. The unclassified (U) phenotype exhibited no expression of CD10, MUC2, MUC5AC, or MUC6. The schema of the phenotypic classification is shown in the Supplementary Figure 1.

**Assessment of DNA integrity**

DNA integrity was assessed by quantitative real-time PCR (qPCR) using the TaqMan RNase P Detection Reagents Kit, and by the FFPE DNA QC Assay on a real-time PCR machine (Thermo Fisher Scientific). Human control genomic DNA (included with TaqMan RNase P Detection Reagents Kit) was serially diluted four times for a five-point standard curve, and the absolute DNA concentrations were determined. The degree of DNA fragmentation was assessed by determining the ratio of the long amplicon (256 bp) to the short amplicon (87 bp), and the RQ (relative quantification) value was used as an indicator of the quality of the genomic DNA.

**Next-generation sequencing**

We performed next-generation sequencing (NGS) for 34 cases, all of which passed the RQ check using a custom 50 gene Ion AmpliSeq panel (Supplementary Figure 2) which was based on the Ion Ampliseq Cancer Hotspot Panel v2 (Thermo Fisher Scientific). Using 10 ng of DNA, library preparation and bar coding were carried out on Ion Chef (Thermo Fisher Scientific), utilizing the Ion AmpliSeq Kit for Chef DL8 (Thermo Fischer Scientific). The products were purified from the other reaction components using Agencourt AMPure XP (Beckman Coulter, Inc., Brea, CA, USA) and then quantified by the qPCR and diluted to 50 pM. Template preparation and chip loading were also performed on the Ion Chef using Ion 510 & Ion 520 & Ion 530 Kit Chef (Thermo Fisher Scientific). A maximum of 34 barcoded samples were used on the Ion 530 chip (Thermo Fisher Scientific). Sequencing was performed on Ion GeneStudio S5 (Thermo Fisher Scientific), utilizing the Variant Caller plugin v.5.10.1.20 which references the COSMIC database. The resulting BAM files were visualized using the Integrative Genomics Viewer (IGV; http://www.broadinstitute.org/igv/). The variant caller files were subjected to further analysis with the Ion Reporter v.5.10 (Thermo Fisher Scientific). The coverage analysis was assessed to evaluate the quality of the sequencing run, using the coverage analysis plug-in v.5.10.0.3. We considered the NGS amplification to be successful if an average minimum of 500 reads or greater was achieved across all target regions and the number of mapped reads was > 15000. DNA from the corresponding non-tumoral tissues was also analyzed as a control. We excluded mutations with < 3% allele frequency. The variants were further annotated with COSMIC (Catalogue of Somatic Mutations in Cancer, http://cancer.sanger.ac.uk/cancergenome/projects/cosmic/) database to provide information on whether those variants have been observed before in the context of any tumor type.
