# Supplementary figures and images for: Gastric epithelial neoplasm of fundic-gland mucosa lineage: proposal for a new classification in association with gastric adenocarcinoma of fundic-gland type
Source: J Gastroenterol. 2021 Jul 15;56(9):814–28. doi: 10.1007/s00535-021-01813-z (PMC8370942; doi:10.1007/s00535-021-01813-z)

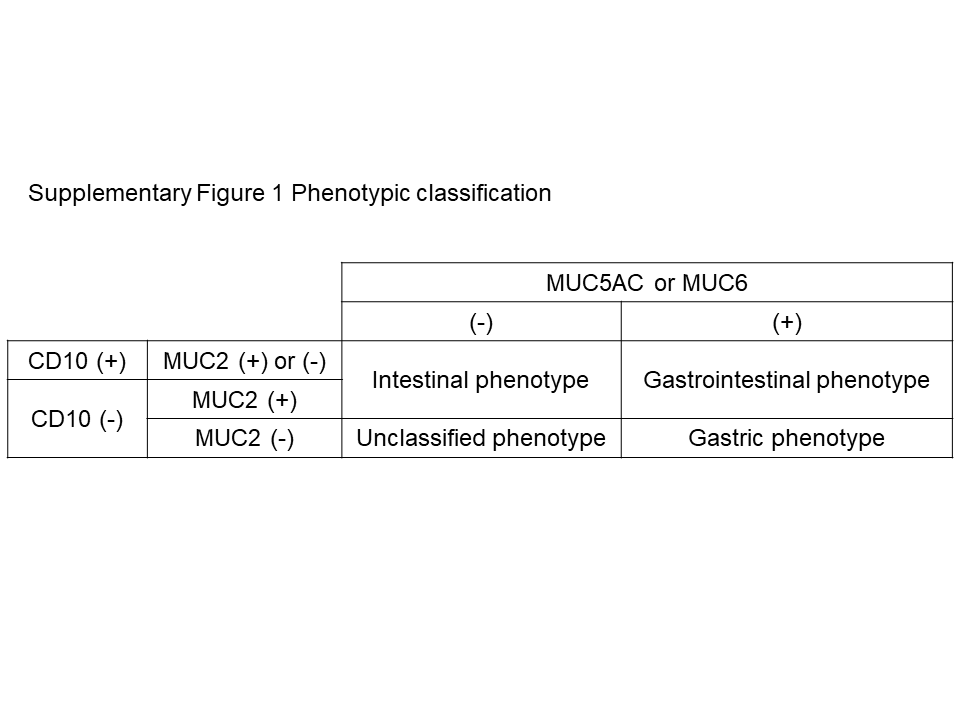

Supplement: Supplementary file 2 — Supplementary file2 Supplementary Fig. 1 Phenotypic classification (TIF 65 KB) [file 535_2021_1813_MOESM2_ESM.tif]

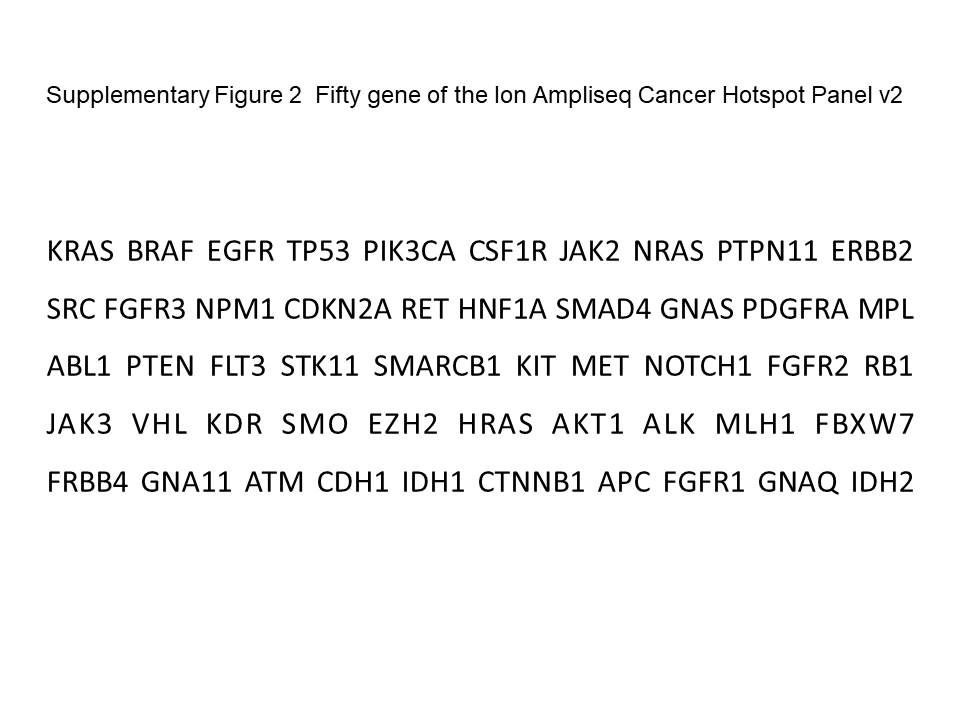

Supplement: Supplementary file 3 — Supplementary file3 Supplementary Fig. 2 Fifty gene of the Ion Ampliseq Cancer Hotspot Panel v2 (TIF 87 KB) [file 535_2021_1813_MOESM3_ESM.tif]

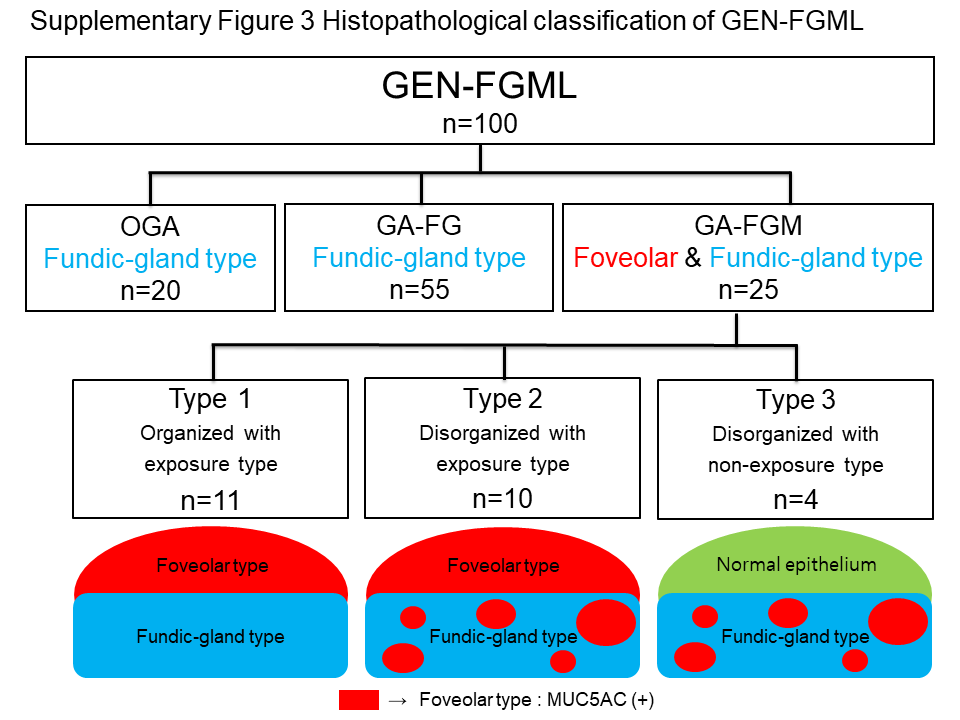

Supplement: Supplementary file 4 — Supplementary file4 Supplementary Fig. 3 Histopathological classification of GEN-FGML (TIF 107 KB) [file 535_2021_1813_MOESM4_ESM.tif]

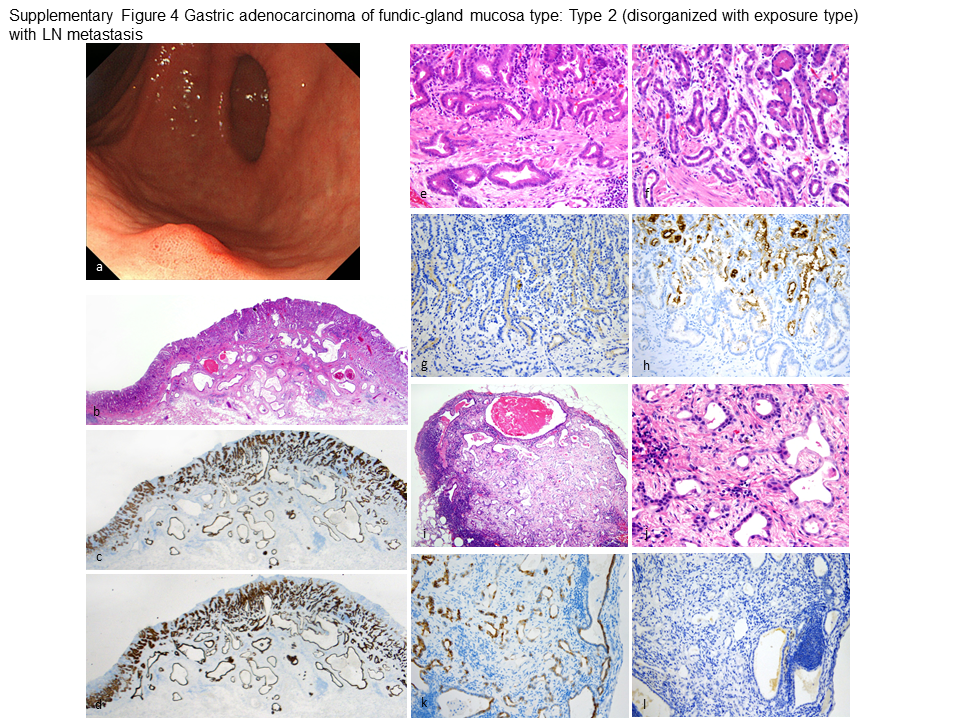

Supplement: Supplementary file 5 — Supplementary file5 Supplementary Fig. 4 Gastric adenocarcinoma of fundic-gland mucosa type: Type 2 (disorganized with exposure type) with LN metastasis. Endoscopic image by white light endoscopy; a The reddish elevated lesion was located at the greater curvature of the lower third of the stomach. The background mucosa had atrophic change. Histological features (b, e, f, i, j); b The surface area of the lesion was composed of foveolar type well-differentiated adenocarcinoma with low-grade atypia. b, e. Adenocarcinoma resembling fundic-gland cells was located at deep area and showed irregular branching and dilatation. f In the middle of the mucosal layer, the tumor cells were composed of highly differentiated columnar cells mimicking fundic-gland cells. i, j. LN metastasis was shown at n#4d. Immunohistochemical results (c ,d, g, h, k, l); c MUC5AC (surface and deep area +), d MUC6 (surface and deep area +), g pepsinogen-I (focally +), h H+/K+-ATPase (focally +). Lymph node k MUC6 (+), l pepsinogen-I (focally +).The layered architecture was destroyed, and tissue construct of foveolar epithelium and fundic gland is collapsed, and tumor is exposed on the surface. Pathological diagnosis; L, 0-IIa, 19x15mm, Adenocarcinoma of fundic-gland mucosa type, T1b/SM2, UL0, Ly0, V0, pHM0, pVM0, pN1 (n#4d:1/4) (TIF 1431 KB) [file 535_2021_1813_MOESM5_ESM.tif]
