# Supplementary material for: Gastric epithelial neoplasm of fundic-gland mucosa lineage: proposal for a new classification in association with gastric adenocarcinoma of fundic-gland type
Source: J Gastroenterol. 2021 Jul 15;56(9):814–28. doi: 10.1007/s00535-021-01813-z (PMC8370942; doi:10.1007/s00535-021-01813-z)
Supplement: Supplementary file 6 — Supplementary file6 (DOCX 13 KB) [file 535_2021_1813_MOESM6_ESM.docx]

| **Supplementary Table 1** Immunohistochemical classification of GEN-FGML | | | |
| --- | --- | --- | --- |
| Antibodies | Marker | OGA and GA-FG | GA-FGM |
| MUC5AC | foveolar epithelial cell | - | + |
| MUC6 | mucous neck cell | + or - | + or - |
| pepsinogen-I  and/or  H^+^/K^+^-ATPase | chief cell  parietal cell | + | + |
| GEN-FGML, gastric epithelial neoplasm of fundic-gland mucosa lineage; OGA, oxyntic gland adenoma; GA-FG, gastric adenocarcinoma of fundic-gland type; GA-FGM, gastric adenocarcinoma of fundic-gland mucosa type. | | | |
