# Supplementary material for: Gastric epithelial neoplasm of fundic-gland mucosa lineage: proposal for a new classification in association with gastric adenocarcinoma of fundic-gland type
Source: J Gastroenterol. 2021 Jul 15;56(9):814–28. doi: 10.1007/s00535-021-01813-z (PMC8370942; doi:10.1007/s00535-021-01813-z)
Supplement: Supplementary file 7 — Supplementary file7 (DOCX 17 KB) [file 535_2021_1813_MOESM7_ESM.docx]

| **Supplementary Table 2** Clinicopathological characteristics, immunohistochemical analysis, and *GNAS* mutation of GEN-FGML (n=100) | |
| --- | --- |
| **Clinicopathological characteristics** | |
| Sex (male : female) | 67 : 33 |
| Age (average: years) | 66.6 (range: 38-87) |
| Therapy | ESD : EMR : OPE= 77 : 11 : 12 |
| Location | U : M : L = 79 : 19 : 2 |
| Morphological classification | protruded : flat/depressed = 63 : 37 |
| Size of tumor (average: mm) | 11.2 (range: 3-85) |
| Depth of invasion (M : SM)  Depth of submucosal invasion (average: μm) | 24 : 76  507.7 (range: 50-4750) |
| Lymphatic invasion | 6%, 6/100 |
| Venous invasion | 6%, 6/100 |
| Horizontal margin | 6%, 6/100 |
| Vertical margin | 3%, 3/100 |
| Lymph node metastasis | 6.3%, 1/16 |
| *H.pylori* infection (n=74) | (+) : 8, (-) : 52, (Eradication) : 14 |
| Survival time (average: months) (n=81) | 23.9 (range: 1-113) |
| Outcome (n=81) | 80 cases: Alive NED, one case died of CVD |
| **Immunohistochemical analysis** | |
| pepsinogen-1 | 100%, 100/100 |
| H^+^/K^+^-ATPase (>focally+) | 80.9%, 72/89 |
| MUC2 | 1.1%, 1/94 |
| MUC5AC | 25%, 25/100 |
| MUC6 | 99%, 99/100 |
| CD10 | 1.1%, 1/94 |
| Phenotypic classification | G : GI = 92 : 2 |
| Chromogranin-A (focally+) | 1.8%, 1/56 |
| p53 overexpression (focally+) | 5.6%, 5/90 |
| Ki-67 labeling index (%) | 6.4% (86 cases) |
| ***GNAS* mutation** | 20.6%, 7/34 |
| GEN-FGML, gastric epithelial neoplasm of fundic-gland mucosa lineage; ESD, endoscopic submucosal dissection; EMR, endoscopic mucosal resection; OPE, operation; U, upper third of the stomach; M, middle third of the stomach; L, lower third of the stomach; M, intramucosal cancer; SM, submucosal cancer; NED, no evidence of disease; CVD, cardiovascular disease; G, gastric phenotype; GI, gastrointestinal phenotype. | |
