# Supplementary material for: Gastric epithelial neoplasm of fundic-gland mucosa lineage: proposal for a new classification in association with gastric adenocarcinoma of fundic-gland type
Source: J Gastroenterol. 2021 Jul 15;56(9):814–28. doi: 10.1007/s00535-021-01813-z (PMC8370942; doi:10.1007/s00535-021-01813-z)
Supplement: Supplementary file 8 — Supplementary file8 (DOCX 15 KB) [file 535_2021_1813_MOESM8_ESM.docx]

| **Supplementary Table 3** Clinicopathological findings of patients who did not meet the curative criteria after endoscopic treatment (n=15) | | | | | | | |
| --- | --- | --- | --- | --- | --- | --- | --- |
| case | Type | Depth of SM invasion | Lymphatic invasion | Venous invasion | Additional surgery | Survival time (months) | Outcome |
| 1 | GA-FG | 800 | - | - | - | 82 | Alive NED |
| 2 | GA-FG | 1100 | - | - | - | 18 | Alive NED |
| 3 | GA-FG | 1400 | - | - | - | 35 | Alive NED |
| 4 | GA-FG | 500 | - | + | Unknown | NA | Unknown |
| 5 | GA-FG | 600 | - | - | - | 1 | Alive NED |
| 6 | GA-FGM (Type 1) | 1500 | - | - | - | 1 | Alive NED |
| 7 | GA-FGM (Type 1) | 700 | - | - | - | 1 | Alive NED |
| 8 | GA-FGM (Type 2) | 1200 | + | - | + | 47 | Alive NED |
| 9 | GA-FGM (Type 2) | 800 | - | - | Unknown | NA | Unknown |
| 10 | GA-FGM (Type 2) | 1000 | - | - | - | 17 | Alive NED |
| 11 | GA-FGM (Type 2) | 400 | + | - | - | 18 | Alive NED |
| 12 | GA-FGM (Type 2) | 400 | + | - | Unknown | NA | Unknown |
| 13 | GA-FGM (Type 2) | 3800 | + | + | Unknown | NA | Unknown |
| 14 | GA-FGM (Type 2) | 1500 | + | + | + | 30 | Alive NED |
| 15 | GA-FGM (Type 2) | 1000 | + | + | + | 1 | Alive NED |
| SM, submucosal; GA-FG, gastric adenocarcinoma of fundic-gland type; GA-FGM, gastric adenocarcinoma of fundic-gland mucosa type; NED, no evidence of disease; NA, not assessed. | | | | | | | |
