# Supplementary material for: Gastric epithelial neoplasm of fundic-gland mucosa lineage: proposal for a new classification in association with gastric adenocarcinoma of fundic-gland type
Source: J Gastroenterol. 2021 Jul 15;56(9):814–28. doi: 10.1007/s00535-021-01813-z (PMC8370942; doi:10.1007/s00535-021-01813-z)
Supplement: Supplementary file 9 — Supplementary file9 (DOCX 20 KB) [file 535_2021_1813_MOESM9_ESM.docx]

| **Supplementary Table 4** Genetic alterations by next-generation sequencing (n=34) | | | | | | |
| --- | --- | --- | --- | --- | --- | --- |
| Case | Type | Gene | Protein | Coding | COSMIC ID | *H.pylori* infection |
| 1 | OGA | (-) |  |  |  | Nagative |
| 2 | OGA | (-) |  |  |  | Nagative |
| 3 | OGA | *GNAS* | p.Arg201Leu | c.602G>T | COSM99221 | NA |
| 4 | GA-FG | *GNAS* | p.Arg201Cys | c.601C>T | COSM27887 | Nagative |
| 5 | GA-FG | (-) |  |  |  | NA |
| 6 | GA-FG | (-) |  |  |  | Nagative |
| 7 | GA-FG | (-) |  |  |  | Nagative |
| 8 | GA-FG | *KRAS* | p.Ala18Asp | c.53C>A | COSM542 | Nagative |
| 9 | GA-FG | *KRAS* | p.Gln61His | c.183A>C | COSM554 | Nagative |
| 10 | GA-FG | (-) |  |  |  | Nagative |
| 11 | GA-FG | *PIK3CA* | p.His1047Tyr | c.3139C>T | COSM774 | Eradication |
| 12 | GA-FG | (-) |  |  |  | Nagative |
| 13 | GA-FG | (-) |  |  |  | Nagative |
| 14 | GA-FG | (-) |  |  |  | Eradication |
| 15 | GA-FG | (-) |  |  |  | Nagative |
| 16 | GA-FG | (-) |  |  |  | Nagative |
| 17 | GA-FG | (-) |  |  |  | Eradication |
| 18 | GA-FG | (-) |  |  |  | Nagative |
| 19 | GA-FG | (-) |  |  |  | Eradication |
| 20 | GA-FG | (-) |  |  |  | Eradication |
| 21 | GA-FG | (-) |  |  |  | Nagative |
| 22 | GA-FG | (-) |  |  |  | Eradication |
| 23 | GA-FG | (-) |  |  |  | Eradication |
| 24 | GA-FG | (-) |  |  |  | Eradication |
| 25 | GA-FG | *GNAS* | p.Arg201His | c.602G>A | COSM27895 | Eradication |
| 26 | GA-FG | *GNAS* | p.Arg201Cys | c.601C>T | COSM27887 | Nagative |
|  |  | *GNAS* | p.Gln227Leu | c.680A>T | COSM27888 |  |
|  |  | *CDKN2A* | p.Met53Thr | c.158T>C | COSM13704 |  |
| 27 | GA-FG | (-) |  |  |  | Nagative |
| 28 | GA-FGM | *GNAS* | p.Arg201Cys | c.601C>T | COSM27887 | Nagative |
| 29 | GA-FGM | (-) |  |  |  | Nagative |
| 30 | GA-FGM | *GNAS* | p.Arg201His | c.602G>A | COSM27895 | Nagative |
| 31 | GA-FGM | (-) |  |  |  | NA |
| 32 | GA-FGM | (-) |  |  |  | Nagative |
| 33 | GA-FGM | *GNAS* | p.Arg201His | c.602G>A | COSM27895 | Eradication |
|  |  | *PIK3CA* | p.Asn345Lys | c.1035T>A | COSM754 |  |
| 34 | GA-FGM | (-) |  |  |  | Eradication |
| OGA, oxyntic gland adenoma; GA-FG, gastric adenocarcinoma of fundic-gland type; GA-FGM, gastric adenocarcinoma of fundic-gland mucosa type; NA, not assessed. | | | | | | |
