# Supplementary material for: Gastric epithelial neoplasm of fundic-gland mucosa lineage: proposal for a new classification in association with gastric adenocarcinoma of fundic-gland type
Source: J Gastroenterol. 2021 Jul 15;56(9):814–28. doi: 10.1007/s00535-021-01813-z (PMC8370942; doi:10.1007/s00535-021-01813-z)
Supplement: Supplementary file 10 — Supplementary file10 (DOCX 19 KB) [file 535_2021_1813_MOESM10_ESM.docx]

| **Supplementary Table 5** Clinicopathological characteristics, immunohistochemical analysis, and genetic alterations of *H.pylori* negative group and *H.pylori* positive or eradicated group | | | |
| --- | --- | --- | --- |
|  | *H.pylori* negative group (n=52) | *H.pylori* positive or eradicated group (n=22) | *P* value |
| **Clinicopathological characteristics** |  |  |  |
| OGA : GA-FG : GA-FGM (Type 1 : Type 2 : Type 3) | 13 : 28 : 11 (8 : 2 : 1) | 5 : 14 : 3 (1 : 1 : 1) | 0.87 |
| Sex (male : female) | 34 : 18 | 13 : 9 | 0.80 |
| Age (average: years) | 64.2 (range: 38-85) | 70.7 (range: 43-87) | <0.05 |
| Therapy | ESD : EMR : OPE= 44 : 7 : 1 | ESD : EMR : OPE= 18 : 3 : 1 | 0.94 |
| Location | U : M : L = 44 : 7 : 1 | U : M : L = 14 : 8 : 0 | 0.15 |
| Morphological classification | protruded : flat/depressed = 40 : 12 | protruded : flat/depressed = 13 : 9 | 0.20 |
| Size of tumor (average: mm) | 9.8 (range: 2-43) | 8.2 (range: 3-44) | 0.46 |
| Depth of invasion (μm) | M : SM = 3 : 36 (GA-FG and GA-FGM)  361.1 (50-1400) µm | M : SM = 0 : 17 (GA-FG and GA-FGM)  347.1 (50-1500) µm | 0.55  0.92 |
| Lymphatic invasion | 1.9%, 1/52 | 4.5%, 1/22 | 0.51 |
| Venous invasion | 1.9%, 1/52 | 4.5%, 1/22 | 0.51 |
| Horizontal margin | 5.8%, 3/52 | 4.5%, 1/22 | 1.00 |
| Vertical margin | 1.9%, 1/52 | 0%, 0/22 | 1.00 |
| Lymph node metastasis | 0%, 0/3 | 0%, 0/3 | 1.00 |
| *H.pylori* infection | (+) : 0, (-) : 52, (Eradication) : 0 | (+) : 8, (-) : 0, (Eradication) : 14 | NA |
| Survival time (average: months) | 25.4 (range: 1-105) (51 cases) | 18.8 (range: 1-104) (21 cases) | 0.41 |
| Outcome | 51 cases: Alive NED | 21 cases: Alive NED | NA |
| **Immunohistochemical analysis** |  |  |  |
| pepsinogen-1 | 100%, 52/52 | 100%, 22/22 | 1.00 |
| H^+^/K^+^-ATPase (>focally+) | 82.4%, 39/47 | 86.4%, 19/22 | 1.00 |
| MUC2 | 2.0%, 1/51 | 0%, 0/22 | 1.00 |
| MUC5AC | 21.6%, 11/51 | 13.6%, 3/22 | 0.53 |
| MUC6 | 98.0%, 50/51 | 100%, 22/22 | 1.00 |
| CD10 | 2.0%, 1/50 | 0%, 0/22 | 1.00 |
| Phenotypic classification | G : GI = 49 : 2 | G : GI = 22 : 0 | 1.00 |
| Chromogranin-A (focally+) | 0%, 0/28 | 0%, 0/11 | 1.00 |
| p53 overexpression (focally+) | 0%, 0/48 | 0%, 0/21 | 1.00 |
| Ki-67 MIB1 LI (%) | 7.0% (46 cases) | 3.8% (21 cases) | <0.05 |
| **Genetic alterations** |  |  |  |
| *GNAS* mutation | 20.0%, 4/20 | 18.2%, 2/11 | 1.00 |
| *KRAS* mutation | 10.0%, 2/20 | 0%, 0/11 | 0.53 |
| *PIK3CA* mutation | 0%, 0/20 | 18.2%, 2/11 | 0.12 |
| *CDKN2A* mutation | 5.0%, 1/20 | 0%, 0/11 | 1.00 |
| OGA, oxyntic gland adenoma; GA-FG, gastric adenocarcinoma of fundic-gland type; GA-FGM, gastric adenocarcinoma of fundic-gland mucosa type; ESD, endoscopic submucosal dissection; EMR, endoscopic mucosal resection; OPE, operation; U, upper third of the stomach; M, middle third of the stomach; L, lower third of the stomach; NA, not assessed; M, intramucosal cancer; SM, submucosal cancer; NED, no evidence of disease; CVD, cardiovascular disease; G, gastric phenotype; GI, gastrointestinal phenotype. | | | |
